# Predictors of the use of psycho-oncological care

**DOI:** 10.1007/s00432-026-06584-9

**Published:** 2026-08-19

**Authors:** J. Schultke, M. Reeh, J.-E. Michaelis, P. Rothe, Y. Hagmayer, F. Braulke

**Affiliations:** 1 Göttingen Comprehensive Cancer Center (G-CCC), University Medical Center Göttingen, Von-Bar-Straße 2/4, 37075 Göttingen, Germany; 2 Georg Elias Müller Institute for Psychology, Georg August University Göttingen, Göttingen, Germany

**Keywords:** Psycho-oncological distress, Cancer patients, Certification, Cancer centers, Psycho-oncological care

## Abstract

**Purpose:**

Psychosocial stressors and mental disorders can negatively affect the success of cancer treatments. Therefore, they should be addressed by target group-oriented psycho-oncological care. This study examined possible predictors of the use of psycho-oncological care.

**Methods:**

Routine data from 5217 patients of a German Comprehensive Cancer Center between 2020 and 2023 were analysed in a retrospective monocentric study. The effects of predictors (gender, age, distance to the center, subjective stress at intake, and tumor stage) on the use of psycho-oncological care (use vs. no use) and on the number of psycho-oncological care sessions lasting at least 20 min were explored using logistic and binomial regression analyses.

**Results:**

Women at younger age, experiencing subjective distress, and patients with advanced-stage cancer were significantly more likely to use psycho-oncological support. Female patients, younger patients, patients who lived closer to the treating centre, patients with subjective distress, and patients at higher tumor stages attended more sessions of psycho-oncological counselling. The analyses also indicated substantial differences between different organ-specific cancer centers.

**Conclusion:**

All patients should be screened for psychological distress and wish for psycho-oncological support by a structured and validated tool. Vulnerable patients, patients at risk, and those, who are hesitant to seek help, could be screened by an interview to reduce barriers.

**Supplementary Information:**

The online version contains supplementary material available at 10.1007/s00432-026-06584-9.

## Introduction

The number of cancer cases has been rising steadily for years, and a further increase is expected in the coming years (World Health Organization 2024). The psychosocial burden and thus the need for psycho-oncological care are high among cancer patients. Previous studies have found a prevalence of 30 to more than 60 percent of significant psychosocial stress during cancer (Goerling et al. 2023; Kendall et al. 2011; Peters et al. 2020; Zabora et al. 2001; Zingler et al. 2025a). In Peters et al. (2020), 65.9% of patients reported a score of at least five and one-third a score of eight or higher on the Distress Thermometer (DT), with the cut-off value for distress being five (Mehnert et al. 2006). More than one-third of patients suffer from comorbid mental disorders during cancer (Mehnert et al. 2014). Common comorbid mental disorders include anxiety disorders, adjustment disorders, and affective disorders (Mehnert et al. 2014). Comorbid mental disorders are associated with a reduced quality of life (Gonzalez-Ling et al. 2022). In addition, there is evidence that distress and mental disorders have a negative impact on the course of cancer treatment (Madhusudhana et al. 2021). Psycho-oncological care during cancer can reduce distress (Blenkiron et al. 2014). Psycho-oncological interventions also have a small to moderate effect in reducing distress and improving quality of life (Faller et al. 2013).

According to the current German S3 guideline, the primary goal of psycho-oncological care is to maintain or improve the subjective quality of life of patients (Working Group of Scientific Medical Societies [AWMF], German Cancer Society [DKG], German Cancer Aid [DKH] 2023). All cancer patients should be screened for distress as early as possible and repeatedly throughout the course of treatment. The wish for psycho-oncological care should be asked for and taken into account, regardless of the experienced distress (AWMF, DKG & DKH 2023). To provide optimal care, it is important to make targeted offers to patients who desire psycho-oncological care or who are experiencing subjective stress (AWMF, DKG & DKH 2023). All certified organ cancer centers have to fulfill all requirements of the German Cancer Society, and resources for psycho-oncological care usually depend on the number of cancer patients treated at the center (AWMF, DKG & DKH 2023).

Therefore, it is important to know, which patients are most likely to request and to frequently use psycho-oncological care. Research on the predictors provides respective insights (Azuero et al. 2014; Cameron et al. 2005; Faller et al. 2017; Tondorf et al. 2018; Zeissig et al. 2015; Zingler et al. 2025a, b).

### State of research on predictors of utilization

Previous studies have examined the effect of gender on subjective stress and the use of psycho-oncological care. Although some studies found no effect of gender on utilization (Azuero et al. 2014; Tondorf et al. 2018), the majority of studies reported that women use psycho-oncological care more frequently (Faller et al. 2017; Zeissig et al. 2015; Zingler et al. 2025a) and report higher distress (Carter et al. 2025; Peters et al. 2020; Zingler et al. 2025a). Goerling et al. (2023) observed that women were more likely to have a positive attitude toward psycho-oncological care than men.

Another frequently studied predictor is age. A large number of studies concluded that younger patients report higher distress levels (Peters et al. 2020) and are more likely to use psycho-oncological support (Azuero et al. 2014; Cameron et al. 2005; Faller et al. 2017; Zeissig et al. 2015). Only a few studies found no respective effect (Blenkiron et al. 2014; Tondorf et al. 2018).

Easy access to psycho-oncological care may also affect its use. Fischl et al. (2023) investigated whether the distance between the place of residence and the treating center has an effect. Contrary to their assumption, distance had no significant influence on the uptake of psycho-oncological care (Fischl et al. 2023). However, to date, no study has investigated whether there is a correlation between distance and the frequency of use.

Former studies on the influence of subjective stress yielded mixed findings. Goerling et al. (2023) found that patients who reported more subjective stress were more likely to use psycho-oncological care, but other predictors had a significantly stronger influence. Zingler et al. (2025a) showed that only 22.6% of patients who reported elevated levels of stress also used psycho-oncological care. Tondorf et al. (2018), demonstrated that 71% of patients who received psycho-oncological care reported elevated levels of stress. Weis et al. (2018) showed higher subjective distress and a more symptoms of anxiety disorders in patients receiving psycho-oncological care compared to those without. Faller et al. (2017) found out that the likelihood of seeking care increases with the severity of depressive symptoms, the severity of anxiety symptoms, or the presence of a known mental disorder.

A higher Union for International Cancer Control (UICC) stage and disease progression were found to be related to an increase in distress (Peters et al. 2020; Zeissig et al. 2015). The UICC stage of the disease is one of the most common criteria of staging cancer. The stage increases depending on the size of the tumor, the involvement of lymph nodes, and the presence of distant metastases (0 = pre-stage to IV = distant metastases present) (Union for International Cancer Control 2025). Although patients with advanced UICC stages report higher levels of distress (Peters et al. 2020), it has not been investigated whether they seek psycho-oncological care more often.

Another predictor that has been investigated is social support. In the study by Faller et al. (2017), less social support was associated with a higher probability of using psycho-oncological care.

The results on socioeconomic status are mixed. In one study, a higher level of education was associated with a higher probability of utilization (Eichler et al. 2023). In contrast, Weis et al. (2018) found that the level of education had no significant effect.

Another predictor examined was the attitude toward psycho-oncological care. A positive attitude had a significant effect on utilization (Faller et al. 2017). In Goerling et al. (2023), a positive attitude toward psycho-oncological care was the strongest predictor of utilization.

Singer et al. (2012) analysed the use of psycho-oncological care in several cancer centres in Germany and found differences between five organ cancer centres: Out of all patients who received at least 30 min of psycho-oncology care, more than 50% were treated within the breast cancer center, compared to only 6.8% treated within the prostate cancer center (Singer et al. 2012).

### Objective

This retrospective single-center analysis aimed to investigate the predictors for the use of psycho-oncological care in patients with different cancer entities in a German Oncology Center certified according to the requirements of the German Cancer Society (Deutsche Krebsgesellschaft, DKG) and Comprehensive Cancer Center (CCC) using structured distress screenings during routine cancer care.

Based on the studies cited above, we expected women to use psycho-oncological care more frequently than men. The same was expected for younger patients and patients experiencing more distress. More frequent use was also assumed with increasing UICC stage.

We explored the distance from the place of residence as a further predictor, although no clear hypothesis could be derived from previous findings. Based on our clinical experience, a negative effect of distance on the use of psycho-oncological care was expected.

## Materials and methods

### Patient cohort

For this retrospective study, routine data of adult cancer patients with an initial diagnosis between 2020 and 2023 who were treated at a German CCC and had at least one psycho-oncological screening were analysed. Data of 10 certified organ cancer centers were included (Breast Cancer Center, Gynecological Cancer Center, Skin Cancer Center, Head and Neck Tumor Center, Lung Cancer Center, Sarcoma Center, Uro-Oncology Center, Gastrointestinal Oncology Center, Center for Hematological Malignancies, Neuro-Oncology Center). Patients who were not assigned to any certified organ cancer center, were grouped separately (Other, n = 30). Some centers became certified during the study period. All patients signed a treatment contract with the institution, and gave their consent to the use of their data for scientific purposes. This study was approved by the local Ethics committee (AZ: 32/10/24).

### Methods

All routine patient data analysed here were obtained from the tumor documentation system (Onkostar®, IT Choice, Karlsruhe, Germany) and were pseudonymized before analysis. The following variables are analysed:

gender (male vs. female), age in years at the time of initial diagnosis, distance from patient´s home to the CCC (calculated from the postal codes of the patient´s home), psycho-oncological screening with date and subjective level of stress, wish for psycho-oncological counselling, duration of psycho-oncological counselling (in minutes), measurement instrument, cancer diagnosis, cancer stage, and the certified organ cancer center the patient was assigned to.

### Screening tools

All Cancer patients at the CCC receive a paper-based questionnaire at least once at initial diagnosis or first contact, and sequentially (usually quarterly) during therapy. Until 2022 the Hornheider screening Inventory (HSI, Strittmatter 2006) was used. Since 2022 the HSI was replaced by the Distress thermometer (DT, Mehnert et al. 2006) due to the recommendations of the S3 guideline (AWMF, DKG & DKH 2023). Both questionnaires contain an additional question whether psychosocial support is wanted or not. Thus, both questionnaires assess subjective distress and the wish for support.

Questionnaires are filled in by patients themselves, checked by psycho-oncologists and documented afterwards manually within the tumor documentation system. Psycho-oncologists contact those patients with screening results above the cut-off value or the documented wish to receive psychosocial support. Patients at risk (e.g., at the transplantation unit) are screened by an experienced psychologist using an interview, and distress is rated on a scale from zero to ten by the psycho-oncologist (according to the DT). This is documented in the tumor documentation system as well.

The HSI has seven items to be rated on a scale from 0 to 2 (total scores from 0 to 14) with a cut-off value of four (Strittmatter 2006). The DT has only one item. A thermometer is visually presented with a scale ranging from zero (no distress) to ten (extreme distress). The threshold for the presence of distress is five (Mehnert et al. 2006).

Psycho-oncologists document each consultation within the tumor documentation system including the date and duration of consultation according to the certification requirements of the German Cancer Society. Whether cancer patients receive psychosocial support outside the CCC (e.g., close at home, during rehabilitation or at public centers for psychosocial support) is not documented.

Based on the available data, two outcome variables were computed for the current set of analyses: First, it was determined whether a patient used psycho-oncological care at all (yes vs. no). Second, the number of psycho-oncological care sessions lasting at least 20 min were analysed. A pragmatic cut-off of 20 min was applied to distinguish psycho-oncological interventions and short contacts. This is consistent with clinical practice, where meaningful psycho-oncological sessions range between 11 and 30 min (Lieb et al. 2025; Singer et al. 2012) with average contact durations around 24.82 min (Lieb et al. 2025). Throughout the text, psycho-oncological care sessions are defined as those lasting at least 20 min. We did not consider the expressed wish for psycho-oncological care as a further outcome variable, as everybody who indicated a wish received an offer.

### Statistic analysis

The data were analysed using the software R, version 4.3.3 (R Core Team 2024). Contrast coding was used for categorical predictor variables, and z-standardization was used for metric predictors. A sum contrast was used for the dichotomous variable gender. Female gender served as the reference. Age was categorized and divided into four categories due to its skewed distribution, with the first category covering a larger age range (18 to 55, 56 to 65, 66 to 75, 76 and above). Polynomial contrast coding was used for age. The distance to the cancer center was calculated from postal codes using the *geosphere* package (Hijmans et al. 2024), and the resulting distances were standardized. Subjective distress was transformed into a categorical variable based on the thresholds published in the literature (four for the HSI, Strittmatter 2006; five for the DT, Mehnert et al. 2006). Since the screening by interview is on a scale 0 to 10, the cut-off value was also set at five.

Screenings for which no value was recorded were assigned to the category *No value*. This category was included because patients could still indicate whether they wished psycho-oncological support even if they did not provide a distress-rating. A sum contrast was used for subjective distress. The characteristic < *cut-off* served as the reference. Since there were differences between screening procedures, we added the screening tool as an additional predictor. Sum contrasts were used and the DT was used as reference. The ordered categorical variable UICC stage (I to IV) was contrast coded with polynomial contrasts.

Linear mixed models with fixed effects for the predictors and a random effect for the organ cancer centers were fitted using the packages *lme4* (Bates et al. 2015) and *glmmTMB* (Brooks et al. 2017). As patients were nested within the certified organ-specific cancer centers, the organ cancer centers served as a grouping variable. A mixed logistic regression was calculated for *the use of psycho-oncological care*. Odds ratios and their 95% confidence intervals were computed as effect estimates. A mixed negative binomial regression was used to investigate the effects of the predictors on the *number of care sessions lasting at least 20 min* was calculated because the distribution of the outcome was skewed to the right. Incidence rate ratios (IRRs) with corresponding 95% confidence intervals were computed as effect estimates. As the screening method had an effect on outcomes, we also ran separate analyses for each screening tool to validate our findings.

## Results

**Fig. 1:**
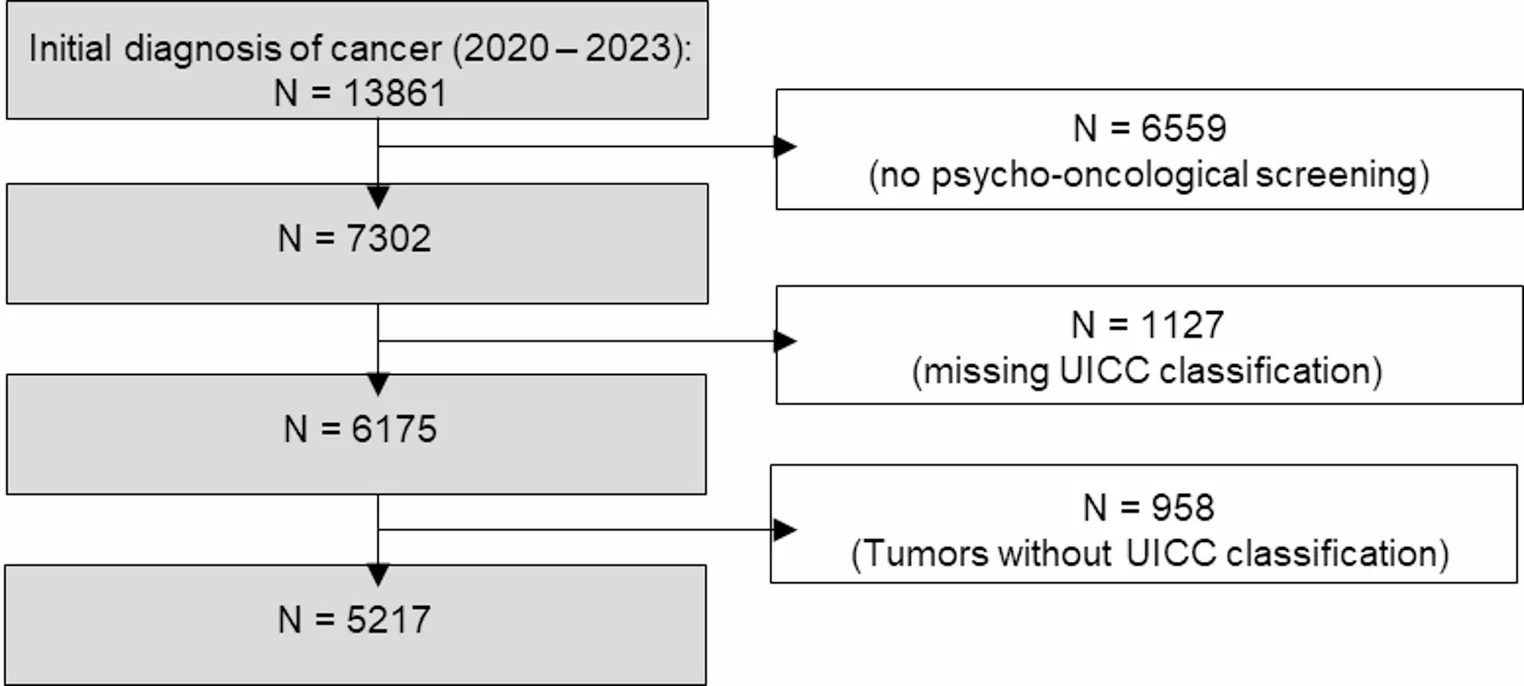
Overview of patients analysed after inclusion and exclusion criteria

For this retrospective study, routine data of 13861 adult cancer patients with initial diagnosis between 2020 and 2023 who were treated at a German CCC were considered. The UICC stage was available for 5217 patients. Patients with hematologic malignancies (N = 787) or neuro-oncological diseases (N = 171) are not classified according to UICC and therefore were excluded. For further 1127 patients the UICC stage was also missing for other reasons (see Fig. 1 for the flow of participants). Thus, data from 5217 patients were analysed.

Of these, 62.2% were male (N = 3245) and 37.8% were female (N = 1972). The median age was 66 years (range 18–101 years). Table 1 shows the patient characteristics. In total, 40.96% of patients (N = 2137) reported subjective distress. For 11.88% no distress value was available, but their wish for psycho-oncological care was recorded.

**Table 1:** Patient characteristics

Predictors	N (%)	Median (range)
Gender	5217 (100.00)	
Male	3245 (62.20)	
Female	1972 (37.80)	
Age (years)	5217 (100.00)	66 (59–73)
18–55	837 (16.04)	
56–65	1617 (30.99)	
66–75	1759 (33.72)	
> 75	1004 (19.24)	
Distance to the Center (kilometers)	5217 (100.00)	30.48 (17.06–45.27)
Organ cancer centers	5217 (100.00)	
Breast	491 (9.41%)	
Gyn	136 (2.61%)	
Skin	533 (10.22%)	
H&N	494 (9.47%)	
Lung	837 (16.04%)	
Sarcoma	10 (0.19%)	
Uro	1541 (29.54%)	
GI	1145 (21.95%)	
Other	30 (0.58%)	
Subjective stress perception	5217 (100.00)	30 (14.29–57.14)
< Cut-off	2460 (47.15)	
> Cut-off	2137 (40.96)	
No value	620 (11.88)	
UICC stage	5217 (100.00)	
I	1213 (23.25)	
II	1418 (27.18)	
III	1346 (25.80)	
IV	1240 (23.77)	

N = absolute frequency of patients, % = relative frequency, range = 25th and 75th. Organ cancer centers abbreviated as follows: Breast = Breast Cancer Center, Gyn = Gynecological Cancer Center, Skin = Skin Cancer Center, H & N = Head and Neck Tumor Center, Lung = Lung Cancer Center, Sarcoma = Sarcoma Center, Uro = Uro-Oncology Center, GI = Gastrointestinal Oncology Center, Other = who were not assigned to any certified organ cancer center. UICC stage: total number of patients with cancers classified according to UICC stages (UICC: Union for International Cancer Control)

### Wish for support

Of the 2137 patients, who had a distress rating about the cut-off, 1596 (74.68%) expressed a wish for psycho-oncological care compared to 996 of 2460 (40.49%), who had a value below the cut-off. Of the 620 patients, who did not provide a distress rating, 382 (61.61%) stated a wish.

### Use of psycho-oncological care

More than half of the patients received psycho-oncological care at the center (N = 2876, 55.13%). There was a high overlap between the wish and the use of care (96.5%, see Table S1 in the Supplement). Table 2 shows the utilization in the individual certified organ cancer centers. Utilization rates were highest in the gynecological cancer center (83.09%), the breast cancer center (76.99%) and lung cancer center (72.88%).

**Table 2:** Use of psycho-oncological care and number of psycho-oncological care in patients according to certified organ cancer centers

		Breast	Gyn	Skin	H & N	Lung	Sarcoma	Uro	GI	Other	N total (%)
A	Yes (%)	378 (76.99%)	113 (83.09%)	172 (32.27%)	316 (63.97%)	610 (72.88%)	4 (40.00%)	621 (40.30%)	647 (56.51%)	15 (50.00%)	2876 (55.13%)
	N (%)	491 (9.41%)	136 (2.61%)	533 (10.22%)	494 (9.47%)	837 (16.04%)	10 (0.19%)	1541 (29.54%)	1145 (21.95%)	30 (0.58%)	5217 (100%)
B	Med (Q1/Q3)	1.0 (0/2)	1.0 (0/2.5)	1.0 (1/2)	1.0 (1/2)	1.0 (1/2)	2.5 (1.75/3.5)	1.0 (0/1)	1.0 (1/2)	1.0 (1/2)	1.0 (1/2)
	Min/Max	(0/15)	(0/26)	(0/41)	(0/19)	(0/26)	(1/5)	(0/18)	(0/24)	(0/4)	(0/41)
	N (% receiving)	378 (13.14%)	113 (3.93%)	172 (5.98%)	316 (10.99%)	610 (21.21%)	4 (0.14%)	621 (21.59%)	647 (22.50%)	15 (0.52%)	2876 (100%)

A = Analysis Utilization, B = Analysis Number of sessions > 20 min. Organ cancer centers abbreviated as follows: Breast = Breast Cancer Center, Gyn = Gynecological Cancer Center, Skin = Skin Cancer Center, H & N = Head and Neck Tumor Center, Lung = Lung Cancer Center, Sarcoma = Sarcoma Center, Uro = Uro-Oncology Center, GI = Gastrointestinal Oncology Center, Other = who were not assigned to any certified organ cancer center. The tables show absolute frequencies and relative frequencies within centers for utilization (A) as well as median (Med), 25th and 75th percentile (Q1/Q3), minimum and maximum values (Min/Max), and absolute and relative frequencies (N) of patients receiving psycho-oncological care for the number of sessions (B)

The results of the statistical analyses are shown in Table 3. Results overall as well as for the individual screening tools are presented. Although there were some differences depending on the screening tool, the overall pattern was similar. The probability of men using psycho-oncological support was significantly lower than that of women (OR 0.73, *p* < .001). Increasing age (linear trend) was a negative predictor of usage (OR 0.67, *p* < .001). Distance from the center had no significant effect on the utilization of psycho-oncology support. Subjective stress had a significant positive effect. Patients who reported a value at or above the cut-off value of the respective instrument were more likely to use psycho-oncological care than patients who reported values below the cut-off (OR 2.35, *p* < .001). Patients for which no specific value was available were significantly less likely to use care (OR 0.77, *p* < .001).

**Table 3:** Predictors of the use of psycho-oncological care and number of psycho-oncological care sessions: Results of the statistical analyses

Predictor	A Utilization				B Number (min. 20 min)			
	Overall N = 5217	HSI N = 1975	DT N = 2034	Interview N = 1208	Overall N = 2876	HSI N = 755	DT N = 970	Interview N = 1151
	OR [95% CI]	OR [95% CI]	OR [95% CI]	OR [95% CI]	IRR [95% CI]	IRR [95% CI]	IRR [95% CI]	IRR [95% CI]
Gender (f)								
Male	0.73*** [0.67, 0.80]	0.68*** [0.59, 0.77]	0.79*** [0.69, 0.90]	0.76 [0.57, 1.02]	0.93*** [0.89, 0.97]	1.00 [0.91, 1.09]	0.89* [0.81, 0.99]	0.91** [0.86, 0.97]
Age (linear trend)	0.67*** [0.57, 0.78]	0.64*** [0.50, 0.82]	0.68** [0.53, 0.86]	0.74 [0.34, 1.61]	0.66*** [0.60, 0.72]	0.61*** [0.51, 0.72]	0.51*** [0.51, 0.75]	0.72*** [0.64, 0.80]
Distance to center (standardized)	0.98 [0.91, 1.04]	0.98 [0.88, 1.06]	0.99 [0.89, 1.11]	0.86 [0.68, 1.10]	0.95** [0.91, 0.99]	0.98 [0.91, 1.05]	0.96 [0.88, 1.05]	0.93* [0.88, 0.98]
Subjective distress (< cut-off)								
> Cut-off	2.35*** [2.12, 2.61]	2.26*** [1.91, 2.68]	2.58*** [2.22, 2.99]	2.74*** [1.76, 4.27]	1.32*** [1.24, 1.40]	1.34*** [1.17, 1.54]	1.06 [0.95, 1.19]	1.53*** [1.41, 1.67]
No value	0.77*** [0.67, 0.89]	0.72** [0.56, 0.92]	0.79* [0.65, 0.96]	0.76 [0.47, 1.23]	0.84 *** [0.76, 0.91]	0.76* [0.60, 0.95]	1.14 [0.98, 1.34]	0.68*** [0.60, 0.78]
UICC stage (linear trend)	1.50*** [1.29, 1.74]	1.63*** [1.30, 2.05]	1.54*** [1.23, 1.91]	0.94 [0.54, 1.63]	1.32 *** [1.21, 1.43]	1.60 *** [1.35, 1.90]	1.18 [1.00, 1.40]	1.30*** [1.16, 1.46]
Screening Tool (DT)								
HSI	0.32*** [0.28, 0.36]				1.06 [1.00, 1.12]			
Interview	8.17*** [6.76, 9.88]				1.31*** [1.25, 1.39]			

Analysis A: mixed logistic regression on the use of psycho-oncological care; N = 5217; random effect of organ cancer centers: variance = 0.30 (SD = 0.55). Analysis B: mixed negative binomial regression on the number of psycho-oncological care sessions lasting at least 20 min; N = 2876 patients who used psychosocial care; random effect of organ cancer centers: variance = 0.02 (SD = 0.15). Predictor values in parentheses = reference value; f = female. OR = odds ratio, IRR = Incidence rate ratios, 95% CI = 95% confidence interval. Significance level: *p* < .05*, *p* < .01**, *p* < .001*** For full results see Table S3 in the Supplement

For patients with tumor stages according to UICC classification, a higher UICC stage was significantly associated with higher utilization (OR 1.50, *p* < .001). As the overall analysis shows, the type of screening also had a significant effect. Screening with the HSI was associated with a significantly lower probability of using psycho-oncological care than screening with the DT (OR 0.32, *p* < .001). Screening by interview was associated with a significantly higher probability of using care than screening by DT (OR 8.17, *p* < .001).

Heterogeneity between the certified organ cancer centers in terms of utilization was evident (see Table 2). A marginal R^2^ of 0.45 indicates that fixed effects of the predictors explained 45% of the variance, whereas the conditional R^2^ of 0.50 demonstrates that inclusion of a random intercept improved model fit. Thus, between center heterogeneity contributed to the overall variability in receiving psycho-oncological care. A small but noticeable proportion of the variation depends on the center in which the patient was treated.

### Number of psycho-oncological care sessions

The number of psycho-oncological care sessions lasting at least 20 min was analysed among patients having at least one contact (N = 2876). The distribution followed a right-skewed distribution with a median of 1 (IQR = 1–2, see Table 2). Hence most patients received between 1 and 2 sessions. The Sarcoma center exhibited the highest number of care sessions (median = 2.5, IQR = 1.75–3.5, see Table 2).The second highest number was found in the Center for hematological malignancies (median = 2, IQR = 1–4), although the center was excluded from further analysis because malignancies are not classified according to UICC (Table S2 in the Supplement), Skin cancer center, head and neck tumor center, lung cancer center GI oncology center and in cancer types without a dedicated certified center demonstrated comparable numbers of sessions (median = 1, IQR = 1–2). In three further organ cancer centers a substantial number of patients only had short consultations (i.e., all centers for which Q1 = 0).

The results of the statistical analyses are shown in Table 3 right hand side. As the screening tool had an impact on the outcomes, results overall and per screening tool are shown. Overall, men received significantly less psycho-oncological care sessions than women (IRR = 0.93, *p* < .001). The number of these longer sessions decreased significantly with increasing age (IRR = 0.66, *p* < .001). The distance from the center had a negative effect (IRR = 0.95, *p* = .014). Patients who lived further away received sessions less frequently at the center. Patients who reported distress above the cut-off of the respective instrument used the longer sessions significantly more often (IRR = 1.32, *p* < .001). The number of sessions also increased with the UICC stage in patients with cancer types classified according to UICC (IRR = 1.32, *p* < .001). Results differed quite substantially between screening tools (see Table 3).

There was only little heterogeneity between the certified organ cancer centers (see Table 2). The fixed effects of the predictors resulted in a marginal R^2^ of 0.19, which indicates predictors explained only 19% of the variance. The conditional R^2^ of 0.22 shows that between center heterogeneity contributed not much to the overall variability in the number of sessions received.

## Discussion

This study aimed to contribute to the strengthening of psycho-oncological care for cancer patients. To this end, predictors of utilization of psycho-oncological care were examined retrospectively based on routine data. We could show that patient characteristics, subjective distress, and characteristics of the disease have a significant influence on the objective utilization. As expected, women were more likely to use psycho-oncological care and also demonstrably more often than men. This in in line with previous studies published (Faller et al. 2017; Zeissig et al. 2015). As expected, utilization decreased with age: Younger patients were more likely to seek psycho-oncological care and received more sessions. This finding also aligns with former published data (Azuero et al. 2014; Cameron et al. 2005; Faller et al. 2017; Zeissig et al. 2015). The study by Singer et al. (2023b) may provide an explanation for this age effect: They found that patients above 65 years of age were less informed about psycho-oncological care than younger patients. The distance to the center had not significant effect on the use of psycho-oncological care in our cohort, confirming similar findings by Fischl et al. (2023). However, the current study also found that patients living closer to their treatment center tended to attend more sessions. This result supports the assumption that patients living closer receive psycho-oncological care more frequently at the center. The psychosocial support network close at home was not part of this analysis. It might be possible, that patients received psychosocial support close to home in addition to or after treatment at the center. This should be addressed in future prospective trials. Our cohort showed, that subjective distress also had a demonstrable effect: Patients with distress above the respective threshold were significantly more likely to use psycho-oncological care and had more sessions. These findings support the results by Peters et al. (2020) and Zeissig et al. (2015). We could demonstrate, that the UICC disease stage also had a significant effect on utilization of psycho-oncology support. As the stage increases, the likelihood of psycho-oncological care increases, as does the number of care sessions. Previous studies did not investigate the association of stage and utilization but found that the stage is related to distress, which is a predictor of the use of psycho-oncological care (Peters et al. 2020; Zeissig et al. 2015).

Unexpectedly, we found an association between the type of screening procedure and utilization. Patients who had direct contact with a psycho-oncologist and were screened by an interview were more likely to receive psycho-oncological care. One explanation might be that the direct personal contact reduces ignorance about and reservations towards psycho-oncological care (cf. Betker et al. 2025). As reported in the study by Goerling et al. (2023), a positive attitude toward psycho-oncology had a strong influence on utilization. Since it is not possible to screen all cancer patients by individual contacts, the German S3 Guideline on Psycho-Oncological Diagnosis, Counseling, and Treatment of Adult Cancer Patients (2023) recommends a structured screening using DT.

Our findings indicated substantial differences between certified organ cancer centers. These differences cannot be fully explained by the predictors considered in this study. Singer et al. (2012) also found differences in utilization between organ cancer centers, but only five centers were considered.

In sum, the results supported all of our hypotheses and were in line with previous studies. The findings extend previous findings by investigating a larger number of patients cared for in a large variety of certified organ cancer centers. With data from 5217 patients over four years, the results are representative of psycho-oncology care at a German CCC. Our findings also provide important insights into subjective wish for and objective use of psycho-oncological care. Of the patients who expressed a desire for psycho-oncological care, 97% received support at the center. More than half of the patients (55.13%) used of psycho-oncological care. This proportion is substantially higher than the proportion of 37%, published by Singer et al. (2012).

Our study also provides new insights into the differences in psycho-oncological care between different organ cancer centers: Significantly more patients received respective care in the gynecological cancer center and the breast cancer center than in the skin cancer center (Table 2). As reported in the study by Singer et al. (2012), substantial variability in utilization was also observed in the present study. Similiary, Singer et al. (2012) found differences across cancer centers, with the highest utilization in breast cancer center (66.7%) and the lowest in the prostate cancer center (6.8%).

The median number of psycho-oncological sessions (i.e., contacts lasting at least 20 min) did not differ significantly between the centers. In almost all centers, 50% of patients received one or none. When considering the small random effect of the organ cancer centers, it must be taken into account that the center of hematologic malignancies with the second highest number of care sessions and one of the highest number of patients receiving more than four long sessions (Table S2) was not included in this analysis due to missing UICC stages. One explanation for these differences might be different durations of hospitalization of cancer patients with regard to the cancer type of treatment modality. Data on the duration of in- and outpatient treatment modalities were not part of this analysis, but should be considered for further prospective trials.

In this study we focused on the actual use of psycho-oncological care depending on the cancer type, cancer stage, patient´s age and gender and the distance from the cancer center to the patient´s home. As the results on the number of longer consultations showed, reaching the center may have an impact for patients as may have the duration of stay.

### Strength and limitations

Due to harmonized national certification requirements for organ cancer centers by the German Cancer Society the different certified organ cancer centers mentioned here are comparable to each other concerning psychosocial support structures and screening requirements. Although the data presented here were based on a retrospective, single-center analysis, they represent a real-world setting of every-day routine care in a large certified German Cancer Center.

There might be a bias in our analyses since organ cancer centers were not certified all at the same time. The present study is a retrospective, single-center analysis of routine data. The number of available variables was limited. Other potentially important predictors of utilization, such as mental health problems, socioeconomic status, social support, knowledge of psycho-oncological care and attitudes toward these services could not be considered (Eichler et al. 2023; Faller et al. 2017; Goerling et al. 2023). Especially, mental health problems may at the same time increase the subjective need for care and impede its usage, e.g., by lack of energy, fatigue, sleep disorders and pain (Schulze et al. 2022). Therefore, these factors should be analysed in future studies. Data on migration background or language barriers were also not available in our study. Limited proficiency in the local language is frequently used as an exclusion criterion in many psycho-oncological studies (Peters et al. 2020; Tondorf et al. 2018; Zingler et al. 2025b). In a multilingual study by Singer et al. (2023a), there was no association between migration background and utilization after adjusting for gender, age, education and patient-relative status. But findings indicate that informational deficits about psycho-oncological care may be more pronounced in specific cultural groups (Singer et al. 2023a). This highlights the importance of further research including migration-related factors. Our analysis was based on binary genders (male and female), therefore, no inferences can be drawn for non-binary patients.

In this study, subjective distress and wish for psycho-oncological care (i.e., subjective need) but not objective need was considered. Objective need may be measured in future studies by considering mental health status and functional impairments in everyday life. Distress was determined based on the first screening. However, distress does not stay constant during treatment or the course of disease. In fact, changes in distress and utilization of care can be observed during the various treatment cycles and phases of the disease (Bergerot and Araujo 2014; Oh and Cho 2020). Future research should therefore investigate changes in the use of psycho-oncological care during the course of the disease and treatment.

When considering the results, it needs to be taken into account that we had no information on whether patients sought psycho-oncological care closer to home. Hence, the predictors found in this study are only predictors of use of psycho-oncological care at the respective cancer center and not of the utilization of this kind of care per se.

Another, potentially limiting factor is that our data cover the period of the coronavirus pandemic. Contact restrictions may also have led to restrictions in the use of psycho-oncological care in the early phase of the pandemic (Dinkel et al. 2021). In addition, values of distress may have been higher than in other years. Pandemic related distress was found to be a predictor of mental disorders such as generalized anxiety disorder and depression in a cohort of breast cancer patients (Swainston et al. 2020).

### Implications for practice

The results of this study are in line with the S3 guideline (AMWF, DKG & DKH 2023) and clearly show that it is advisable to offer psycho-oncological care to patients with scores on a standardized screening tool that indicate subjective distress. In addition, it is important to consider that direct contact with a psycho-oncologist was associated with more frequent utilization, indicating that there might be additional barriers to an uptake when patients are merely requested to fill in a screening tool. Since distress screening through personal interviews is not possible for all patients in large clinics, interprofessional team meetings and exchanges with other health care professionals (e.g. physicians, oncology nurses) are highly relevant to identify patients in need, who are reluctant to state their need. It might be advisable to screen vulnerable and patients at risk (e.g. patients in stem cell treatment, with advanced and metastatic cancer) by interview.

To reduce stigma (Schulze et al. 2022) and improve access to psycho-oncological support, men and elderly adults should be specifically targeted and provided with tailored information. Patients with higher UICC stage disease should also be specifically targeted, as physical limitations may impede their ability to actively seek support.

To screen for distress, valid screening procedures should be used, as recommended in the S3 guideline. The DT is currently the only validated tool. Cut-offs based on empirical studies should be used to identify patients in distress.

## Conclusion

This study showed that the use of psycho-oncological care is more prevalent among women, younger patients, those with subjective stress, and those with a higher UICC stage disease. It also showed that the use of psycho-oncological support varies between different organ cancer centers within a comprehensive cancer center. Future studies should investigate these differences in more detail in a multi-center, multifunctional prospective setting, including outreach partner. In addition, more focus should be given to vulnerable and patients at risk, who may not seek psycho-oncological support for themselves.

## Supplementary Information

Below is the link to the electronic supplementary material.

Supplementary Material 1

Supplementary Material 2

Supplementary Material 3

## Data Availability

Data availability All data generated or analysed during this study are included in this published article [and its supplementary information files].
