# Supplementary material for: Predictors of the use of psycho-oncological care
Source: J Cancer Res Clin Oncol. 2026 Aug 19;152(8):165. doi: 10.1007/s00432-026-06584-9 (PMC13498528; doi:10.1007/s00432-026-06584-9)
Supplement: Supplementary file 1 [file 432_2026_6584_MOESM1_ESM.docx]

Table S1

Self-reported wish for psycho-oncological care and received care

|  |  | **Requested** | |  |
| --- | --- | --- | --- | --- |
|  |  | **Yes** | **No** | **N** |
| **Received** | **Yes** | 2870 (96.50%) | 6 (0.27%) | 2876 (55.13%) |
|  | **No** | 104 (3.50%) | 2237 (99.73%) | 2341 (44.87%) |
|  | **N** | 2974 (57.01%) | 2243 (42.99%) | 5217 (100%) |

Note: Received = Utilization of psycho-oncological care, Requested = wish for support (measured using screening instruments). N = absolute frequency of patients, % = relative frequency.
