# Supplementary material for: Predictors of the use of psycho-oncological care
Source: J Cancer Res Clin Oncol. 2026 Aug 19;152(8):165. doi: 10.1007/s00432-026-06584-9 (PMC13498528; doi:10.1007/s00432-026-06584-9)
Supplement: Supplementary file 2 [file 432_2026_6584_MOESM2_ESM.docx]

Table S2

Use of psycho-oncological care and number of psycho-oncological sessions in patients according to certified organ cancer centers

|  |  | **Hem** | **Neuro** |
| --- | --- | --- | --- |
| **A** | **Yes (%)**  **N** | 527 (66.96%)  787 | 132 (77.19%)  171 |
| **B** | **Med (Q1/Q3)** | 2.0 (1/4) | 1.0 (0/2) |
|  | **Min/Max** | (0/39) | (0/17) |
|  | **N** | 527 (13.42%) | 132 (3.36%) |

Note: A = Analysis Utilization, B = Analysis Number of sessions > 20 minutes. Organ cancer centers abbreviated as follows: Hem = Center for Hematological Neoplasms, Neuro = Neuro-Oncology Center. The tables show absolute frequencies and relative frequencies within centers for utilization (A) as well as median (Med), 25^th^ and 75^th^ percentile (Q1/Q3), minimum and maximum values (Min/Max), and absolute and relative frequencies (N) of patients receiving psycho-oncological care for the number of sessions (B).
