# Supplementary material for: Predictors of the use of psycho-oncological care
Source: J Cancer Res Clin Oncol. 2026 Aug 19;152(8):165. doi: 10.1007/s00432-026-06584-9 (PMC13498528; doi:10.1007/s00432-026-06584-9)
Supplement: Supplementary file 3 [file 432_2026_6584_MOESM3_ESM.docx]

Table S3

Predictors of the use of psycho-oncological care and number of psycho-oncological care sessions: Full results of the statistical analyses.

|  | **A**  **Utilization** | | | | **B**  **Number of sessions (min. 20 minutes)** | | | |
| --- | --- | --- | --- | --- | --- | --- | --- | --- |
|  | **Overall**  **N = 5217** | **HSI**  **N = 1975** | **DT**  **N = 2034** | **Interview**  **N = 1208** | **Overall**  **N = 2876** | **HSI**  **N = 755** | **DT**  **N = 970** | **Interview**  **N = 1151** |
| **Predictor** | B (SE) | B (SE) | B (SE) | B (SE) | B (SE) | B (SE) | B (SE) | B (SE) |
| Intercept | 0.98 (0.20)*** | -0.27 (.19) | 0.07 (.27) | 3.86 (.49)*** | 0.35 (.06)*** | 0.33 (.11)** | 0.08 (.09) | 0.58 (.07)*** |
| **Gender** (f)  Male | -0.31 (.04)*** | -0.39 (.07)*** | -0.24 (.07)*** | -0.27 (.15) | -0.08 (.02)*** | -0.003 (.05) | -0.11 (.05)* | -0.09 (.03)** |
| Age  linear trend  quadratic trend  cubic trend | -0.41 (.08)***  0.004 (.08)  0.10 (.06) | -0.44 (.12)***  -0.19 (.11)  0.13 (.09) | -0.39 (.12)**  0.09 (.11)  0.08 (.09) | -0.30 (.39)  0.60 (.33)  -0.01 (.25) | -0.41 (.05)***  0.04 (.04)  0.08 (.04)* | -0.50 (.09)***  0.18 (.08)*  0.10 (.07) | -0.50 (.10)***  -0.10 (.09)  0.07 (.07) | -0.33 (.06)***  -0.01 (.06)  0.07 (.05) |
| **Distance to center**  (standardized) | -0.02 (.03) | -0.04 (.05) | -0.01 (.06) | -0.15 (.12) | -0.05 (.02)** | -0.02 (.04) | -0.04 (.04) | -0.07 (.03)* |
| **Subjective distress**  (<cut-off)  >Cut-off No value | 0.85 (.05)***  -0.26 (.07)*** | 0.82 (.09)***  -0.33 (.13)** | 0.95 (.08)***  -0.24 (.10)* | 1.01 (.23)***  -0.27 (.24) | 0.27 (.03)***  -0.18 (.05)*** | 0.30 (.07)***  -0.28 (.12)* | 0.06 (.06)  0.13 (.08) | 0.43 (.04)***  -0.38 (.10)*** |
| **UICC stage**  linear trend  quadratic trend  cubic trend | 0.40 (.08)***  -0.18 (.07)*  -0.02 (.07) | 0.49 (.12)***  -0.02 (.11)  -0.03 (.10) | 0.43 (.11)***  -0.32 (.11)**  0.01 (.10) | -0.06 (.28)  -0.37 (.33)  0.005 (.37) | 0.28 (.04)***  0.003 (.04)  -0.08 (.04)* | 0.47 (.09)***  0.01 (.08)  0.01 (.08) | 0.16 (.09)  0.16 (.08)  -0.16 (.08)* | 0.26 (.06)***  -0.12 (.06)  -0.05 (.06) |
| **Screening Tool**  (DT)  HSI  Interview | -1.14 (.06)***  2.10 (.10)*** |  |  |  | 0.06 (.03)  0.27 (.03)*** |  |  |  |

Note: Analysis A: mixed logistic regression on the use of psycho-oncological care; N = 5217; random effect of organ cancer centers: variance = 0.30 (SD = 0.55). Analysis B: mixed negative binomial regression on the number of psycho-oncological care sessions lasting at least 20 minutes; N = 2876 patients who used psychosocial care; random effect of organ cancer centers: variance = 0.02 (SD = 0.15). Predictor values in parentheses = reference value; f = female. B = unstandardized regression coefficient, SE = Standard error regression coefficient;. Significance level: p < .05*, p < .01**, p<.001***
